# Two-Dimensional MoS_2_ Field-Effect Biosensor for Highly Sensitive Detection of Cardiac Troponin I

**DOI:** 10.1021/acsami.5c05963

**Published:** 2025-05-16

**Authors:** Yung-Hsin Huang, Yung-Hsuan Chen, Evan Darius, Hui-Fang Shi, Chao-Hui Yeh, Ju-Yin Hsu, Keng-Ku Liu

**Affiliations:** † Department of Biomedical Engineering and Environmental Sciences, 34881 National Tsing Hua University, Hsinchu 300044, Taiwan; ‡ Department of Electrical Engineering, National Tsing Hua University, Hsinchu 300044, Taiwan; § Institute of Electronics Engineering, National Tsing Hua University, Hsinchu 300044, Taiwan; ∥ Center for Nanotechnology, Materials Science and Microsystem, National Tsing Hua University, Hsinchu 300044, Taiwan; ⊥ College of Semiconductor Research, National Tsing Hua University, Hsinchu 300044, Taiwan; # 63423 National Taiwan University Hospital Hsinchu Branch, Hsinchu 300001, Taiwan

**Keywords:** MoS_2_, plasmonic nanomaterials, biosensors, cardiovascular disease, cardiac troponin I

## Abstract

Two-dimensional (2D) materials, particularly transition metal dichalcogenides (TMDs), have gained considerable research attention in electronics and biosensing due to their exceptional electrical and physical properties. In this work, we report a molybdenum disulfide (MoS_2_) field-effect-based biosensor for sensitive, selective, and label-free detection of cardiac troponin I (cTnI), a key biomarker for acute myocardial infarction (AMI). To enhance biorecognition efficiency, yolk–shell-structured plasmonic nanoparticles were synthesized and conjugated with anti-cTnI antibodies before being immobilized on the MoS_2_ channel surface. The resulting biosensor demonstrated high sensitivity with a limit of detection as low as 2.66 pg/mL. Selectivity tests confirmed its excellent specificity, effectively distinguishing cTnI from other interfering biomarkers. The integration of 2D MoS_2_ with yolk–shell nanomaterials provides a highly promising platform for rapid and precise AMI diagnostics.

## Graphical abstract

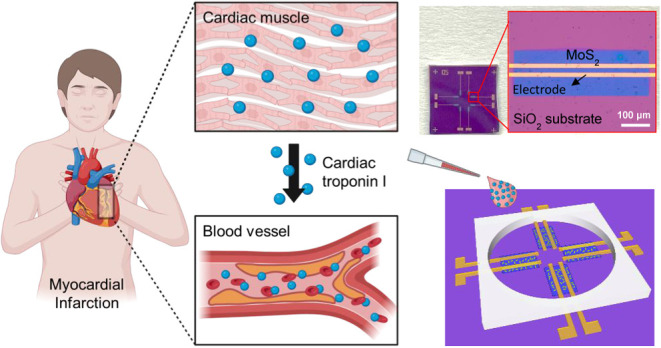

## Introduction

In recent years, cardiovascular disease has emerged as a growing concern within public health systems worldwide.^1^ Among its most severe manifestations, acute myocardial infarction (AMI), characterized by the occurrence of myocardial necrosis due to acute obstruction of blood flow and coronary ischemia, is a leading consequence of coronary artery disease.^2^ Given that cardiovascular diseases account for nearly one-third of deaths, early and accurate diagnosis is crucial for reducing both morbidity and mortality.^1^ Therefore, ultrasensitive and reliable cardiac biomarker quantification is highly essential for effective diagnosis and management.^3^ To facilitate early diagnosis of cardiovascular diseases, various cardiac biomarkers are utilized as indicators to evaluate the risk of patients suspected of having acute coronary syndromes who might develop AMI.^4^ Cardiac troponin I (cTnI), a biomolecule with molar mass approximately 23.8 kDa, is widely used as a biomarker to detect myocardial damage in patients with AMI due to its significant cardiac specificity.^4,5^ As the concentration of cTnI increases within a few hours after the onset of AMI, reaching its maximum level within the next 24 h, it is essential and urgent to reinforce the biosensing platform for timely and precise detection.^1,6,7^

Two-dimensional (2D) materials are at the forefront of materials research due to their exceptional electrical, optical, and electrochemical properties.^8–13^ These unique characteristics provide them with unprecedented potential in electronics, biosensing, and energy applications.^14–18^ For biosensing applications, there is a growing focus on understanding the interfacing interaction between nanomaterials and biological molecules, which is driving significant advancements in this field.^19–21^ Among the various approaches for the fabrication of 2D materials-based biosensors, electrical biosensors have attracted considerable attention for their label-free detection, high sensitivity, rapid response, and portability.^22–24^ In particular, atomically layered 2D transition metal dichalcogenides (TMDs), such as molybdenum disulfide (MoS_2_), molybdenum diselenide (MoSe_2_), tungsten disulfide (WS_2_), and tungsten diselenide (WSe_2_), have been demonstrated to be effective for the next-generation field-effect transistor (FET) biosensors due to their unique electrical and physical properties.^16,21,24,25^ On account of their exceptional properties and large surface area, 2D TMDs materials hold significant potential for biosensing applications.

In this work, we report MoS_2_ field-effect-based biosensors for sensitive and label-free detection of cTnI. Specifically, MoS_2_ serves as the channel material in the field-effect biosensor, providing a highly sensitive platform for electrical signal transduction. Meanwhile, yolk–shell-structured plasmonic nanomaterials were synthesized and subsequently conjugated with anti-cTnI antibodies. These antibody-functionalized nanomaterials were immobilized on the MoS_2_ channel surface to enhance the surface area of the MoS_2_ biosensors and enable the specific detection of target cardiac biomarkers. Utilizing this 2D MoS_2_ field-effect biosensing platform, only a small sample volume of 20 μL is required for testing. Our MoS_2_ biosensors demonstrated high sensitivity, achieving a limit of detection (LOD) with a concentration as low as 2.66 pg/mL of cTnI. Furthermore, selectivity studies with various interfering proteins confirmed that the MoS_2_ biosensors exhibit excellent selectivity for cTnI, meeting the requirements for early AMI diagnosis.

## Results and Discussion

Figure 1 illustrates the integration of yolk–shell plasmonic nanoparticles with MoS_2_ as a biosensor for cardiac biomarker sensing. The chemical vapor deposition (CVD) method was used for the growth of 2D MoS_2_ nanomaterials (see the Experimental Section). The thickness of the as-synthesized MoS_2_ thin layers can be determined using an optical microscope, and the atomic force microscopy (AFM) image revealed a thickness of approximately 3.8 nm (Figures 2a,b). In the photoluminescence (PL) spectrum of MoS_2_, a prominent peak can be observed at the wavelength of 663 nm (Figure 2c). Additionally, the Raman spectrum of MoS_2_ revealed two characteristic peaks at wavenumbers approximately 379 and 402 cm^–1^, which correspond to the *E*_2*g*_^1^ and *A*_1*g*_, respectively (Figure 2d).^26^ The as-synthesized MoS_2_ was further analyzed via X-ray photoelectron spectroscopy (XPS). The Mo binding energy levels of 228.6 and 231.7 eV correspond to the Mo 3d_5/2_ and Mo 3d_3/2_, respectively (Figure 2e). A binding energy level of 225.8 eV can be observed with a relatively weak peak intensity, which can be identified as S 2s due to the Mo–S bonding. The binding energies of 161.5 and 162.6 eV can also be observed in the XPS spectrum, which corresponded to the S 2p_3/2_ and S 2p_1/2_, respectively (Figure 2f).

**Figure 1:**
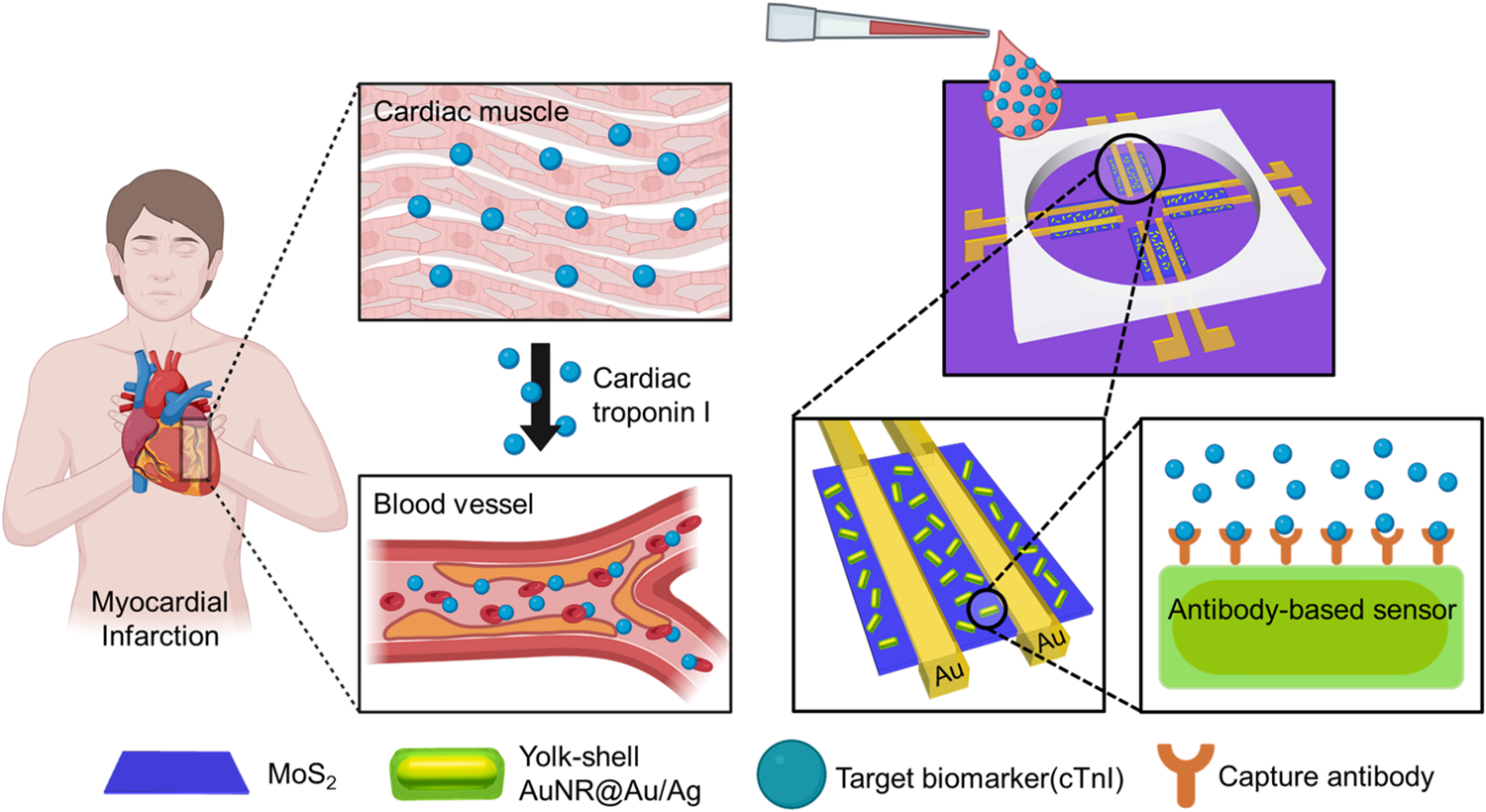
Schematic illustration of the MoS_2_ biosensor for cardiac troponin I sensing.

**Figure 2:**
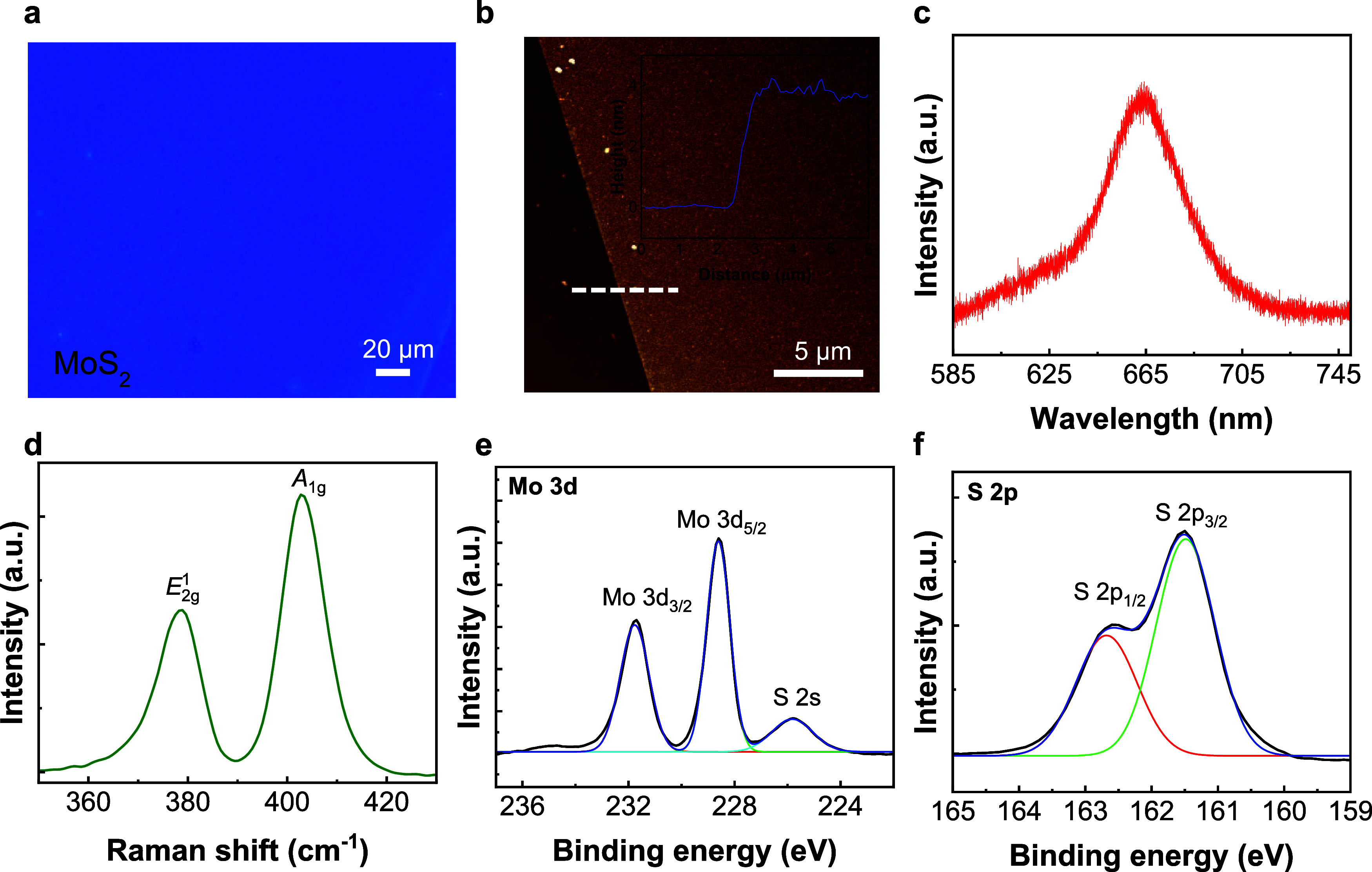
(a) Optical micrograph of the as-synthesized MoS_2_. (b) AFM image of the as-synthesized MoS_2_. The inset shows the height profile measured along the dashed line. (c) PL spectrum of the as-synthesized MoS_2_. (d) Raman spectrum of the as-synthesized MoS_2_. (e) XPS spectrum of Mo 3d and S 2s of the as-synthesized MoS_2_. (f) XPS spectrum of S 2p of the as-synthesized MoS_2_.

After successfully preparing 2D MoS_2_, yolk–shell-structured plasmonic nanomaterials were synthesized. A large number of plasmonic nanomaterials on the MoS_2_ channel surface will enhance the surface area and increase the available sites for the biorecognition elements on MoS_2_ biosensors, ultimately elevating the probe density of the biosensors.^27^ These high-density probes will be beneficial for sensitively detecting specific cardiac biomarkers. A two-step procedure was employed for the synthesis of yolk–shell nanoparticles.^28,29^ The procedure started with the growth of gold nanorods (AuNRs), which served as the cores of the yolk–shell-structured plasmonic nanoparticles (see Supporting Information for details). The size and shape of the as-synthesized AuNRs can be observed using a transmission electron microscope (TEM), and the edge length and width were measured to be 52.2 ± 4.0 and 16.4 ± 1.7 nm, respectively (Figures 3a and S1). The aqueous solution of silver nitrate (AgNO_3_) was added as a precursor of silver, using ascorbic acid as the reducing agent and hexadecyltrimethylammonium chloride (CTAC) as the capping agent, into the AuNRs suspension. A uniform thin layer of Ag was deposited on the surface of AuNRs, forming AuNR core-Ag shell (AuNR@Ag) nanomaterials (Figure 3b). Their average length and width determined from the TEM image were 64.1 ± 4.0 and 28.2 ± 1.7 nm, respectively (Figure S2). A galvanic replacement reaction was employed to transform the solid structure of AuNR@Ag into porous and yolk–shell-structured plasmonic nanomaterials. An aqueous HAuCl_4_ solution was put in the AuNR@Ag suspension, resulting in the yolk–shell-structured plasmonic nanoparticles (Figure 3c). The average dimensions were also determined from the TEM image, which displayed a length of 63.2 ± 3.6 nm and a width of 32.7 ± 1.6 nm (Figure S3). Ultraviolet (UV)–vis-NIR spectra of AuNRs, AuNR@Ag, and yolk–shell AuNR@Au/Ag were collected (Figure 3d). The longitudinal and transverse plasmon resonance wavelengths of the AuNRs dispersed in aqueous solutions are 785 and 510 nm, respectively. Four plasmon resonance bands can be observed at wavelengths of 341, 392, 444, and 582 nm in the spectrum of AuNR@Ag. In addition, the UV–vis-NIR spectrum of yolk–shell AuNR@Au/Ag shows four plasmon resonance bands at wavelengths of 348, 389, 471, and 616 nm. The energy-dispersive X-ray spectroscopy (EDX) elemental mapping results revealed the bimetallic composition of gold and silver within the yolk–shell AuNR@Au/Ag (Figure 3e-h).

**Figure 3:**
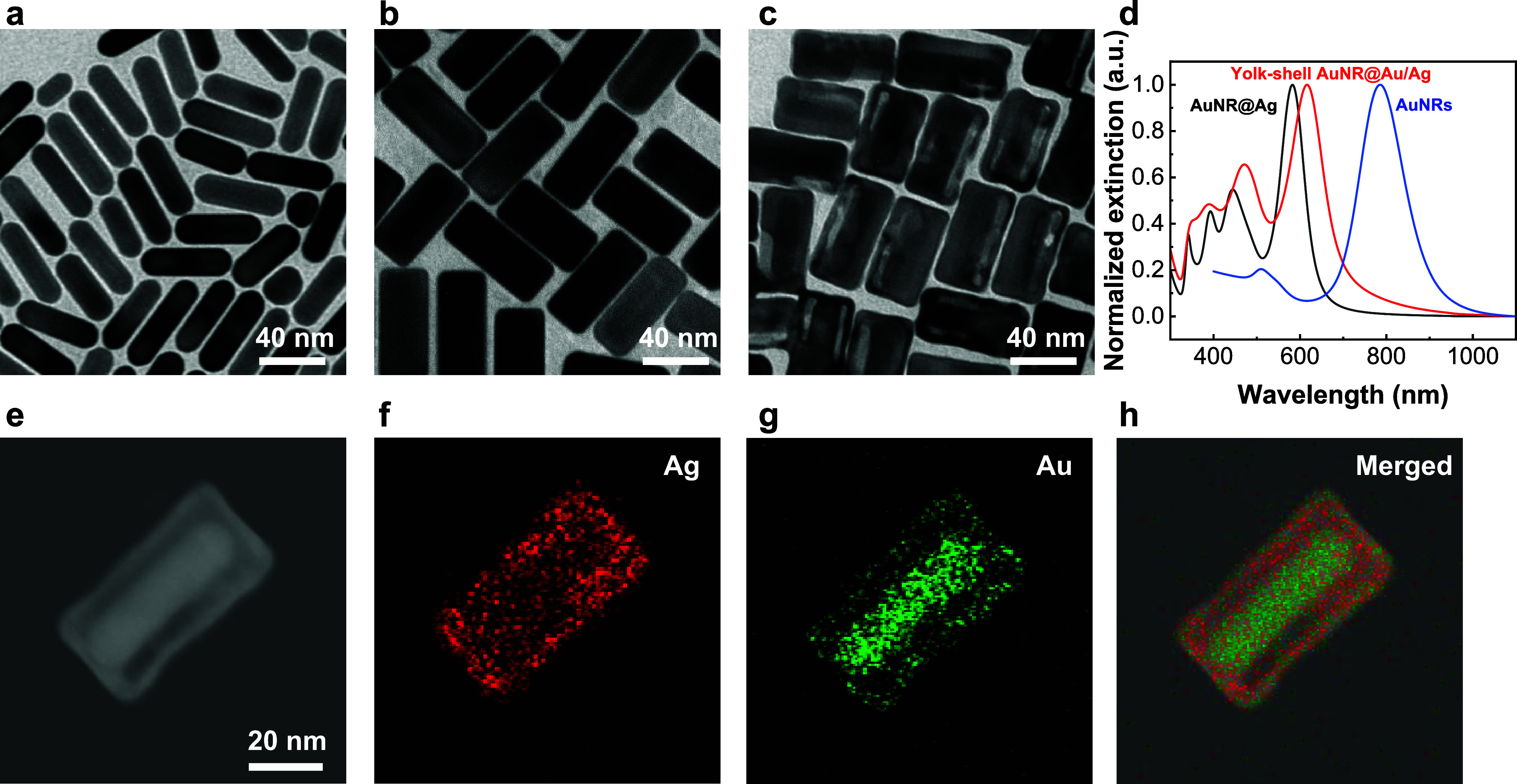
Typical TEM images of (a) AuNRs, (b) AuNR@Ag, and (c) yolk–shell AuNR@Au/Ag. (d) Representative extinction spectra of nanoparticles. (e) HAADF-STEM image and the corresponding EDX elemental mapping of (f) Ag, (g) Au, and (h) merged image of yolk–shell AuNR@Au/Ag.

Following the synthesis and characterization of nanomaterials as building blocks for biosensors, we moved forward with the fabrication of 2D MoS_2_ biosensors. The channel length of these biosensors was designed to be 20 μm, and we utilized a standard lithography-based manufacturing process for their construction. A total of eight biosensors were arranged on a biosensing chip with a size of 1 × 1 cm^2^, and the sensing area in the biosensor was close to the center area of the biosensing chip (Figure 4a). The output characteristics (*I*_DS_–*V*_DS_ curves) of the n-type MoS_2_ device are shown in Figure 4b. The transfer characteristics (*I*_DS_-*V*_GS_ curves) of the MoS_2_ device in logarithmic (black curve) and linear (blue curve) scales were measured at a drain voltage of 1 V (Figure 4c). The on/off ratio of the n-type MoS_2_ device was calculated to be 3.46 × 10^4^. After successful preparation of the MoS_2_ device, we proceeded with the surface functionalization of the MoS_2_ device. The cTnI antibodies were conjugated onto yolk–shell AuNR@Au/Ag via the 1-(3-(dimethylamino)­propyl)-3-ethylcarbodiimide hydrochloride (EDC)/*N*-hydroxysuccinimide (NHS) method (see the details in the Experimental Section). After the conjugation of cTnI antibodies, a red shift of 9 nm was observed in the LSPR spectrum (Figure 4d and Figure S4). Next, cTnI antibody-modified yolk–shell AuNR@Au/Ag nanostructures were adsorbed on the 2D MoS_2_ channel surface via electrostatic interaction using poly­(sodium-4-styrenesulfonate) (PSS) (Figure 4e, please see Experimental Section and Supporting Information for the detailed procedure). The output characteristics of the MoS_2_ device were measured before (red) and after (blue) the adsorption of these nanostructures (Figure 4f). From the output characteristics, the drain current was observed to be decreased after the adsorption of cTnI antibody-modified yolk–shell AuNR@Au/Ag nanostructures. This decrease in current may be attributed to the charge transfer and accumulation effect induced by the cTnI antibody-modified yolk–shell AuNR@Au/Ag nanostructures.^27^ The MoS_2_ biosensor, prepared with the adsorption of cTnI antibody-modified yolk–shell AuNR@Au/Ag nanostructures on the channel surface of the MoS_2_ device, is ready for detection of the target biomarker cTnI.

**Figure 4:**
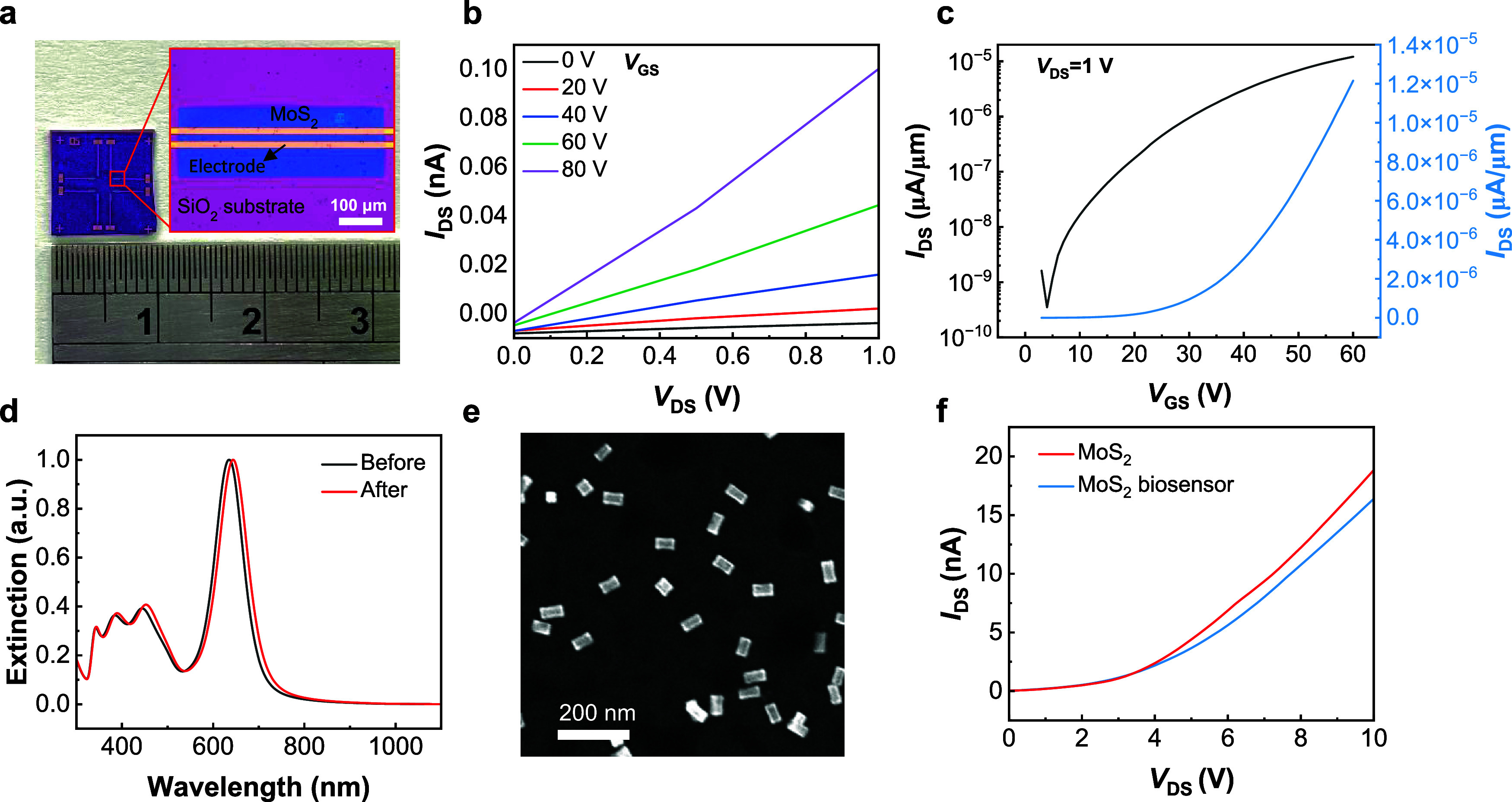
(a) Photograph of the MoS_2_ biosensor. The inset shows an optical micrograph of the sensing area in the biosensor. (b) *I*_DS_–*V*_DS_ curves of the MoS_2_ FET device with the back-gate voltage varying from 0 to 80 V. (c) *I*_DS_–*V*_GS_ curve and the corresponding logarithmic curve of the MoS_2_ FET device. *V*_DS_ was fixed at 1 V. (d) UV–vis-NIR spectra of yolk–shell AuNR@Au/Ag before and after cTnI-antibody conjugation. (e) Representative SEM image shows the cTnI-antibody-conjugated yolk–shell AuNR@Au/Ag on MoS_2_. (f) *I*_DS_–*V*_DS_ curves of the MoS_2_ FET device before (red) and after (blue) the adsorption of cTnI-antibody-conjugated yolk–shell AuNR@Au/Ag. *V*_GS_ is 0 V.

To access the sensing performance of the MoS_2_ biosensor, we tested a 1× Tris-buffered saline (TBS) solution (pH 7.4) spiked with cTnI at various concentrations ranging from 0 to 100,000 pg/mL. Figure 5a shows the *I*_DS_–*V*_DS_ curves of the MoS_2_ biosensor as a function of the cTnI concentration. As the cTnI protein concentration increases, a significant decrease in the current is observed. This behavior is due to the molecular gating effect of the negatively charged cTnI protein, i.e., p-doping, which decreased the electron carrier concentration in MoS_2_.^22,27^ During the sensing experiments, the sensing response is employed to evaluate the sensitivity of the MoS_2_ biosensor, which is defined as ((*I*_0_ – *I*_f_)/*I*_0_) × 100%, where *I*_0_ and *I*_f_ represent the currents in the *I*_DS_–*V*_DS_ curves before and after cTnI detection, respectively. As shown in Figure 5b, the MoS_2_ biosensor can detect a cTnI concentration as low as 10 pg/mL, which is below the typical cTnI levels found in human blood.^7,30^ The calibration curve indicates a strong correlation between the concentration of cTnI and the response of the MoS_2_ biosensor, with a coefficient of determination (*R* ^2^) of 0.993. Additionally, notable differences in response were observed after incubation in 1× TBS containing cTnI at concentrations of 0 and 10 pg/mL, indicating the exceptional sensitivity of the MoS_2_ biosensor for detecting low concentrations of target biomarkers (Figure S5). The LOD was calculated to be 2.66 pg/mL (average signal obtained from the blank sample plus three times its standard deviation). Furthermore, we performed experiments to compare the binding capacities of our biosensors (i.e., anti-cTnI/yolk–shell AuNR@Au/Ag/MoS_2_) with those fabricated via direct antibody immobilization (i.e., anti-cTnI/MoS_2_). For the preparation of directly immobilized biosensors, anti-cTnI antibodies were immobilized onto MoS_2_ using a thiol-based functionalization method. The anti-cTnI/MoS_2_ biosensors were then exposed to 1× TBS containing cTnI at a concentration of 10 pg/mL. Compared to the response observed from the anti-cTnI/yolk–shell AuNR@AuAg/MoS_2_ biosensor under identical conditions (exposure to cTnI at a concentration of 10 pg/mL), the signal obtained from the directly immobilized anti-cTnI/MoS_2_ biosensors was significantly lower (Figure S6 in the Supporting Information). These results clearly demonstrate that yolk–shell AuNR@Au/Ag substantially enhances the sensing performance of the MoS_2_ biosensor. To verify the specificity, the MoS_2_ biosensors were tested with two nontarget biomarkers, C-reactive protein (CRP, 10 ng/mL) and myoglobin (250 ng/mL). As shown in Figure 5c, the response of the biosensor to cTnI (73.55 ± 6.8%) was significantly higher than its responses to CRP (7.58 ± 0.9%) and myoglobin (4.74 ± 1.0%). Furthermore, the selectivity of the biosensors was evaluated in complex media with and without cTnI. The complex medium was composed of various biomolecules found in the human body within normal levels, including hemoglobin (15 g/dL), albumin (4 g/dL), glucose (75 mg/dL), uric acid (5 mg/dL), CRP (10 ng/mL), and myoglobin (250 ng/mL) in 1× TBS. When exposed to the complex medium without cTnI, the biosensor showed a response of approximately 9.45 ± 0.4% (Figure 5d). In contrast, in the presence of cTnI (10 ng/mL), the response increased significantly to 73.65 ± 2.9%, demonstrating the remarkable specificity of the MoS_2_ biosensors.

**Figure 5:**
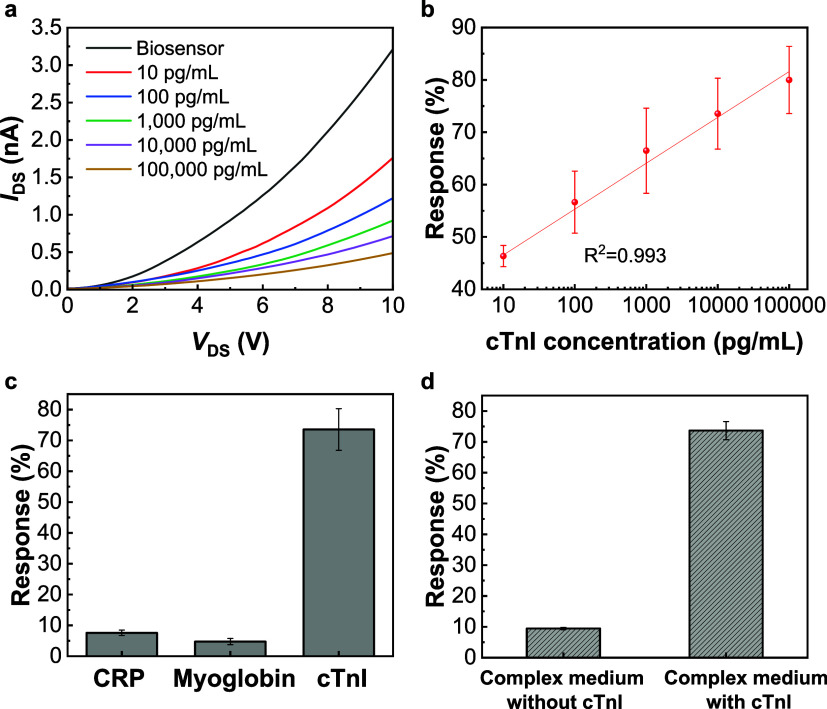
(a) *I*_DS_–*V*_DS_ curves of the MoS_2_ biosensor as a function of the cTnI concentration. *V*_GS_ is 0 V. (b) Response as a function of the cTnI concentration. (c) Response demonstrates its specificity in detecting cTnI compared to two other interfering proteins, CRP and myoglobin. (d) Response of the sensor after exposure to the complex medium without and with cTnI.

## Conclusions

In summary, we have successfully developed a highly sensitive and selective 2D MoS_2_ field-effect biosensing platform for the label-free detection of cTnI, which is a crucial biomarker for acute myocardial infarction. By integrating yolk–shell-structured plasmonic nanomaterials with MoS_2_, the surface area and biorecognition efficiency were significantly enhanced, achieving a remarkable LOD as low as 2.66 pg/mL. The biosensor demonstrated a strong correlation between cTnI concentration and response, with a high coefficient of determination (*R* ^2^ = 0.993). Furthermore, specificity studies confirmed minimal cross-reactivity with nontarget biomarkers, reinforcing the suitability of the biosensor for early and precise AMI diagnosis.

## Experimental Section

### Chemical Vapor Deposition of MoS_2_

Two-dimensional MoS_2_ was synthesized by using the chemical vapor deposition method. First, the MoO_3_ powder (around 3 mg) was placed in a quartz boat in the center of the furnace. The S powder (0.5 g) was placed in a separate quartz boat in the upper stream of the furnace. The SiO_2_/Si substrate was cleaned using a piranha solution, followed by a sonication process in nanopure water for 10 min. The clean SiO_2_/Si substrate was placed face down above the quartz boat containing MoO_3_ powder. The gas flow (80 sccm of Ar) was introduced into the chamber, and the pressure was controlled at 50 Torr. The center zone of the furnace was heated to 750 °C and kept there for 10 min.

### Yolk–Shell AuNR@Au/Ag-cTnI Antibody Conjugate

Yolk–shell AuNR@Au/Ag-cTnI antibody conjugate was obtained using an EDC/NHS method.^31,32^ Please see the Supporting Information for the detailed procedure.

### Adsorption of Nanoparticle-cTnI Antibody Conjugate on MoS_2_

Please see the Supporting Information for the detailed procedure.

### Biosensing Test

During the sensing experiments, cTnI protein was spiked in a 1× TBS solution (pH 7.4), and 20 μL of the sample was incubated in the sensing area of the MoS_2_ biosensor for 1h. After incubation, the biosensor was rinsed with 1× TBS and nanopure water and then dried using nitrogen gas. The electrical experiments were conducted with a Keysight B1500A and Keithley 4200 analyzers. The sensor response is defined as ((*I*_0_ – *I*_f_)/*I*_0_) × 100%, where *I*_0_ and *I*_f_ represent the currents in the *I*_DS_–*V*_DS_ curves before and after cTnI detection, respectively, at a source-drain voltage (*V*_DS_) of 8 V.

### Characterization Techniques

UV–vis-NIR experiments were performed with a Shimadzu UV-1900 spectrophotometer. SEM experiments were conducted with a JEOL JSM-7610F instrument. TEM images were collected with a JEOL JEM-2100 instrument. EDX spectroscopy is affiliated with the TEM instrument. AFM experiments were conducted with a Bruker Dimension ICON. XPS experiments were conducted with a high-resolution electron spectrometer (ULVAC-PHI). Raman and PL spectra were obtained by using a Horiba iHR-550 Raman spectrometer with a 532 nm laser.

## Supplementary Material



## Notes

Materials; synthesis of plasmonic nanomaterials; size distribution of plasmonic nanomaterials; functionalization of cTnI antibodies, and response of the MoS_2_ biosensor (PDF)
