# Supplementary material for: Two-Dimensional MoS2 Field-Effect Biosensor for Highly Sensitive Detection of Cardiac Troponin I
Source: ACS Appl Mater Interfaces. 2025 May 16;17(21):30740–6. doi: 10.1021/acsami.5c05963 (PMC12123573; doi:10.1021/acsami.5c05963)
Supplement: Supplementary file 1 [file am5c05963_si_001.pdf]

Supporting Information

Two-Dimensional MoS_2 Field-Effect Biosensor for Highly Sensitive Detection of Cardiac Troponin I

Yung-Hsin Huang[1], Yung-Hsuan Chen[1], Evan Darius[1], Hui-Fang Shi[1], Chao-Hui Yeh[2,3,4,5], Ju-Yin Hsu[6], and Keng-Ku Liu[1]*

[1.] Department of Biomedical Engineering and Environmental Sciences, National Tsing Hua University, Hsinchu 300044, Taiwan

[2.] Department of Electrical Engineering, National Tsing Hua University, Hsinchu 300044, Taiwan

[3.] Institute of Electronics Engineering, National Tsing Hua University, Hsinchu 300044, Taiwan

[4.] Center for Nanotechnology, Materials Science and Microsystem, National Tsing Hua University, Hsinchu 300044, Taiwan

[5.] College of Semiconductor Research, National Tsing Hua University, Hsinchu 300044, Taiwan

[6.] National Taiwan University Hospital Hsinchu Branch, Hsinchu 300001, Taiwan

*To whom correspondence should be addressed: kkliu@mx.nthu.edu.tw

Materials

Molybdenum (VI) oxide, sulfur powder, gold chloride trihydrate, cetyltrimethylammonium bromide, sodium borohydride, silver nitrate, ascorbic acid, poly(sodium-4-styrene sulfonate), hemoglobin, myoglobin, albumin, glucose, and uric acid were purchased from Sigma-Aldrich. Hexadecyltrimethylammonium chloride, 1-(3-Dimethylaminopropyl)-3-ethylcarbodiimide hydrochloride, and N-hydroxysuccinimide were purchased from TCI. Thiol PEG acetic acid was purchased from JenKem Technology. Troponin I antibody and Troponin I protein were purchased from Fitzgerald.

Synthesis of Au Nanorods

Au nanorods were synthesized by using a seed-mediated method.[1,2] Seed solution was synthesized by adding 0.6 mL of an ice-cold $NaBH_4$ (10 mM) solution into the solution containing 0.25 mL of $HAuCl_4$ (10 mM) and 9.75 mL of CTAB (0.1 M) under vigorous stirring (800 rpm) at room temperature. The color of the seed solution changed from yellow to brown. Subsequently, the growth solution was prepared by mixing 5 mL of $HAuCl_4$ (10 mM), 95 mL of CTAB (0.1 M), 1 mL of $AgNO_3$ (10 mM), and 0.55 mL of ascorbic acid (0.1 M) in succession. The solution was homogenized by gentle shaking. To the colorless solution, 0.12 mL of freshly prepared seed solution was added and kept undisturbed in the dark for 14 h. Before use, the AuNR solution was centrifuged twice at 8000 rpm for 10 min to remove excess CTAB and re-dispersed in nanopure water.

Synthesis of AuNR@Ag

4 mL of twice-centrifuged AuNR (extinction around 2.0) and 8 mL of CTAC (20 mM) were mixed at 60 °C. After stirring for 20 min, 1.6 mL of $AgNO_3$ (2 mM), 2 mL of CTAC (20 mM), and 0.8 mL of ascorbic acid (0.1M) were added under stirring at 60 °C for 4 h.

Synthesis of Yolk-Shell AuNR@Au/Ag

Yolk-shell nanomaterials were synthesized by transforming the Ag shell of AuNR@Ag into the porous shell of Au/Ag via a galvanic replacement reaction. The as-synthesized AuNR@Ag solution was centrifuged at 8,000 rpm for 10 min and re-dispersed in CTAC solution (20 mM). An aqueous solution of $HAuCl_4$ (0.5 mM) was injected into the

AuNR@Ag solution at a rate of 0.5 mL/min under magnetic stirring until the desired LSPR wavelength was achieved. The as-synthesized yolk-shell AuNR@Au/Ag solution was centrifuged at 8,000 rpm for 10 min and re-dispersed in nanopure water.

Preparation of Yolk-Shell AuNR@Au/Ag-cTnI Antibody Conjugate

Yolk-shell AuNR@Au/Ag-cTnI antibody conjugate was prepared by an EDC/NHS approach as reported previously.[3, 4] Briefly, 37.5 µL of SH-PEG-COOH (20 µM), 150 µL of EDC (5 µM), 60 µL of NHS (12.5 µM), and 202.5 µL of nanopure H_2O were mixed and shaken for 1 h at room temperature to activate PEG. After 1 h of shaking, 50 µL of 10x PBS (pH 7.4) was added. Then, 10 µL of cTnI antibody (0.5 µg/mL) was added and shaken for 2 h at room temperature. After 2 h shaking, the mixture was centrifuged using a centrifugal filter (10 kDa) at a rate of 7000 rpm for 10 min, followed by washing with H_2O three times, and re-dispersed in 500 µL of H_2O. The solution was stored at -20 °C before use. For the 1 mL of twice-centrifuged yolk-shell AuNR@Au/Ag (extinction around 2.0), SH-PEG-antibody (10 µL each time) was added into the yolk-shell AuNR@Au/Ag until there is no redshift anymore in the LSPR wavelength.

Adsorption of Yolk-Shell AuNR@Au/Ag-cTnI Antibody Conjugate on MoS_2

The MoS_2 on the silicon substrate was immersed in an aqueous solution of PSS (0.5% w/w) for 45 min, followed by rinsing with nanopure water and drying with nitrogen gas. The yolk-shell AuNR@Au/Ag-cTnI antibody conjugate solution was centrifuged and re-dispersed in nanopure water. PSS-treated MoS_2 on the silicon substrate was incubated with the yolk-shell AuNR@Au/Ag-cTnI antibody conjugate solution at 4 °C overnight.

Conjugation of cTnI Antibody on MoS_2

For the preparation of anti-cTnI antibodies directly immobilized on MoS_2, the SH-PEG-anti-cTnI antibody (10 ng/mL) was incubated on the surface of MoS_2 for 3 h, followed by rinsing with PBS and drying with nitrogen gas.

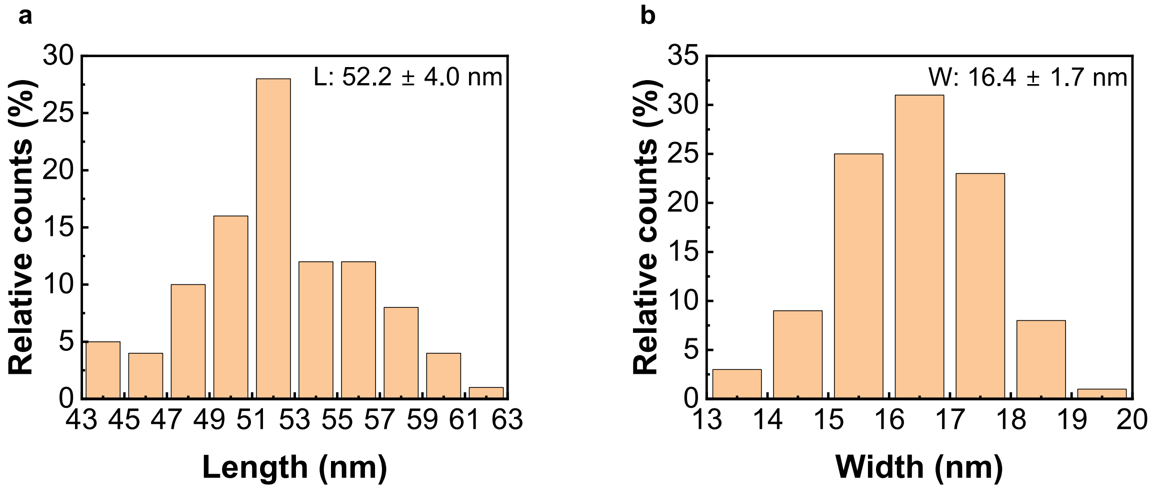

Figure S1. The histogram shows the size distribution of the AuNRs. (a) Length of AuNRs. (b) Width of AuNRs.

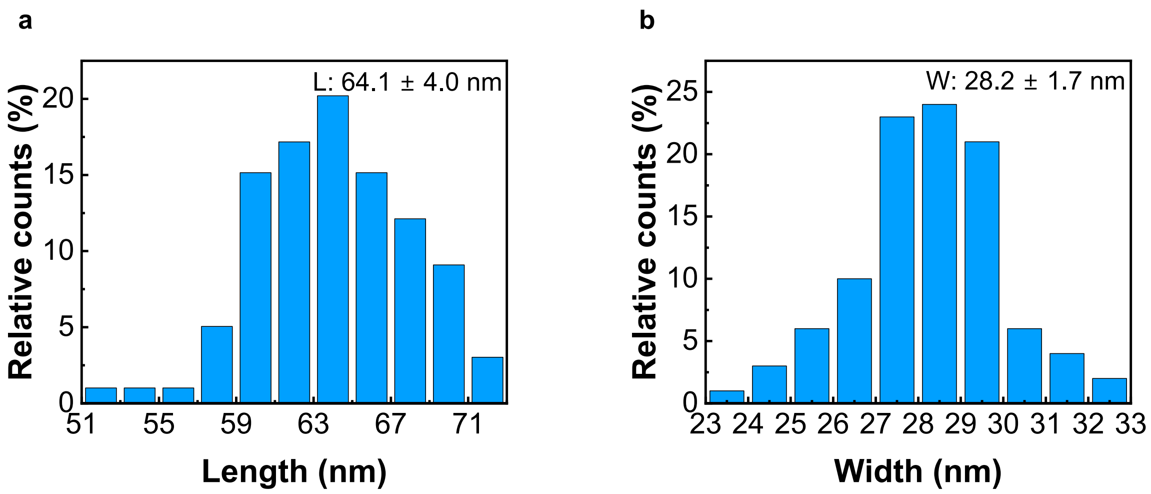

Figure S2. The histogram shows the size distribution of the AuNR@Ag. (a) Length of AuNR@Ag. (b) Width of AuNR@Ag.

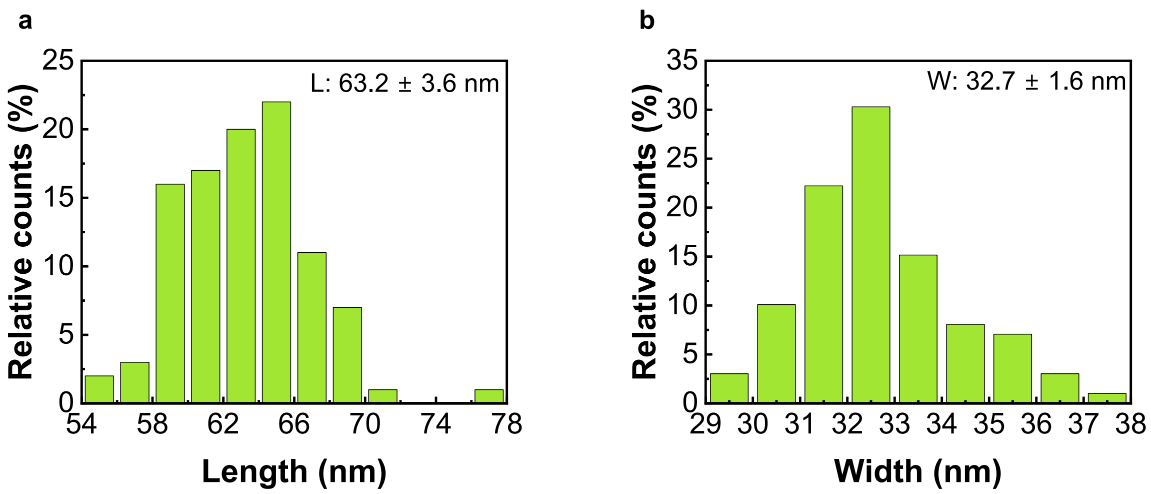

Figure S3. The histogram shows the size distribution of the yolk-shell AuNR@Au/Ag. (a) Length of yolk-shell AuNR@Au/Ag. (b) Width of yolk-shell AuNR@Au/Ag.

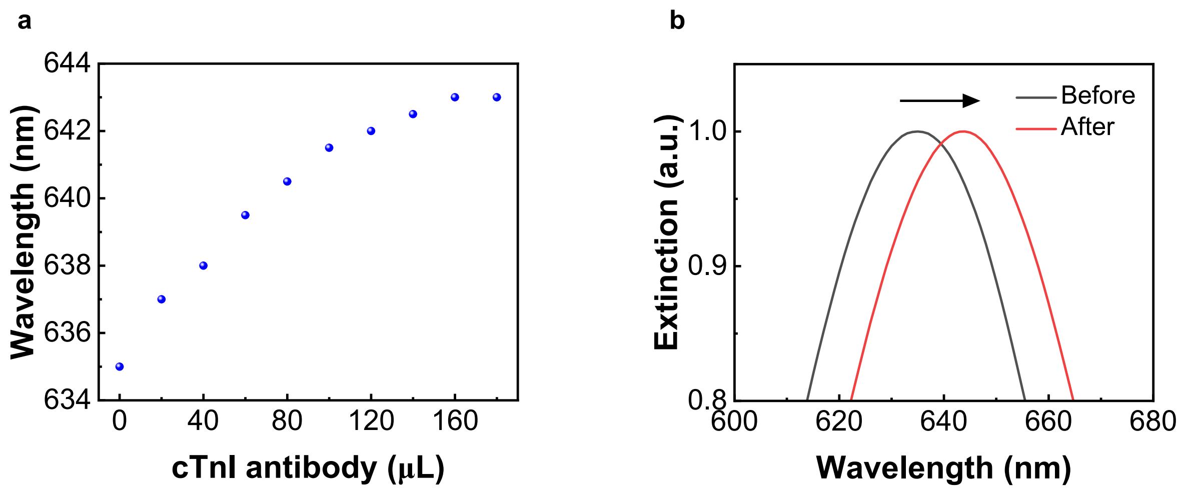

Figure S4. The functionalization of cTnI antibodies on the surface of yolk-shell AuNR@Au/Ag. (a) The LSPR wavelength of yolk-shell AuNR@Au/Ag vs the amount of cTnI antibody added to the yolk-shell AuNR@Au/Ag suspension. (b) The LSPR wavelength of yolk-shell AuNR@Au/Ag before (black line) and after (red line) the addition of cTnI.

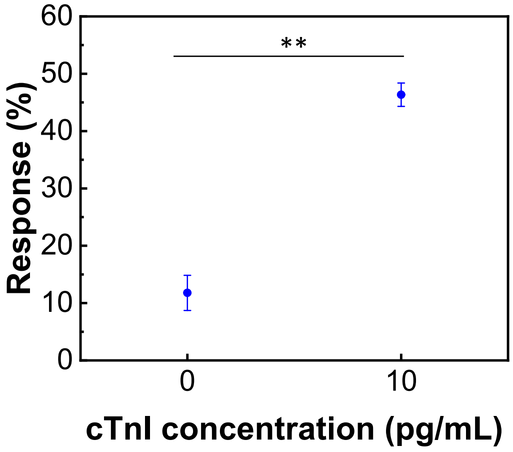

Figure S5. The response of the MoS_2 biosensor after incubation in 1X TBS with cTnI at the concentrations of 0 and 10 pg/mL. n=3, data are mean ± standard deviation. ** $p < 0.01$ by Welch's t-test.

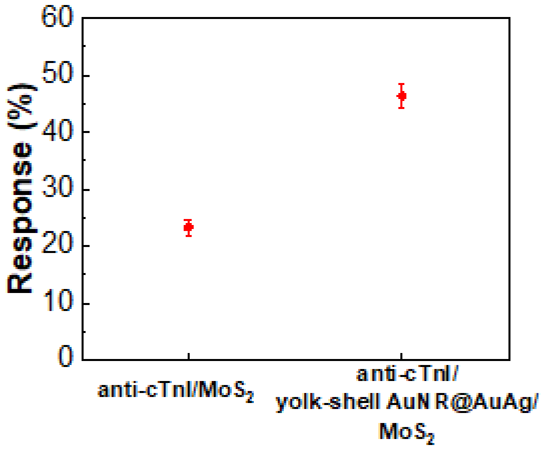

Figure S6. The comparison of the sensing performance between the anti-cTnI/MoS_2 biosensor and the anti-cTnI/yolk-shell AuNR@AuAg/MoS_2 biosensor after incubation in 1X TBS with cTnI at a concentration of 10 pg/mL. n=3, data are mean ± standard deviation.

References

(1) Huang, X.; Neretina, S.; El-Sayed, M. A. Gold Nanorods: From Synthesis and Properties to Biological and Biomedical Applications. *Advanced Materials* **2009**, *21* 4880-4910.
(2) Orendorff, C. J.; Murphy, C. J. Quantitation of Metal Content in the Silver-Assisted Growth of Gold Nanorods. *The Journal of Physical Chemistry B* **2006**, *110* 3990-3994.
(3) Lu, Y.-C.; Darius, E.; Lien, M.-C.; Yeh, I. H.; Shi, H.-F.; Huang, Y.-H.; Chen, C.-H.; Chen, H.-W.; Su, C.-Y.; Hsu, R.-Y.; Liu, K.-K. Two-Dimensional Cs2AgBiBr6-Based Biosensor for Selective and Sensitive Detection of Cardiac Biomarker Troponin I. *ACS Applied Nano Materials* **2023**, *6* 23022-23028.
(4) Yeh, I. H.; Shi, H.-F.; Darius, E.; Lien, M.-C.; Lu, Y.-C.; Wang, C.; Liu, K.-K. Plasmonic biochips with enhanced stability in harsh environments for the sensitive detection of prostate-specific antigen. *Journal of Materials Chemistry B* **2024**, *12* 1617-1623.
